# Laparoscopic RFA with splenectomy for hepatocellular carcinoma

**DOI:** 10.1186/s12957-016-0954-x

**Published:** 2016-07-27

**Authors:** Kunpeng Hu, Purun Lei, Zhicheng Yao, Chenhu Wang, Qingliang Wang, Shilei Xu, Zhiyong Xiong, He Huang, Ruiyun Xu, Meihai Deng, Bo Liu

**Affiliations:** 1 Department of General Surgery, The Third Affiliated Hospital, Sun Yat-sen University, Guangzhou, 510000 China; 2 Department of Gastrointestinal Surgery, The Third Affiliated Hospital, Sun Yat-sen University, Guangzhou, 510000 China; 3 Department of General Surgery, Affiliated Hospital of Jiangnan University, Wuxi, 214000 China; 4 Department of Hepatobiliary Surgery, The Third Affiliated Hospital, Sun Yat-sen University, Guangzhou, 510000 China

**Keywords:** Hepatocellular carcinoma, Hypersplenism, Laparoscopy, Ablation, Splenectomy, Endoscopic variceal ligation

## Abstract

**Background:**

The treatment of hepatocellular carcinoma (HCC) is complicated and challenging because of the frequent presence of cirrhosis. Therefore, we propose a novel surgical approach to minimize the invasiveness and risk in patients with HCC, hypersplenism, and esophagogastric varices.

**Methods:**

This was a retrospective study carried out in 25 patients with HCC and hypersplenism and who underwent simultaneous laparoscopic-guided radio-frequency ablation and laparoscopic splenectomy with endoscopic variceal ligation. Tumor size was restricted to a single nodule of <3 cm. Characteristics of the patients (cirrhosis etiology, liver function, tumor size, spleen size), surgery (complications, blood loss, time of stay), and follow-up (recurrence and survival) were examined.

**Results:**

Mean operative time was 128 ± 18 min. Mean blood loss was 206 ± 57 mL. Length of stay was 7.0 ± 1.5 days. Mean total costs were 8064 USD. Cytopenia and thrombocytopenia recovered quickly after surgery. No procedure was converted to open surgery. Two patients showed worsening liver function after surgery, three patients showed worsening of ascites, and five patients suffered from portal vein thrombosis. The 1-year tumor-free survival was 78.8 %, and the 21-month tumor-free survival was 61.4 %. According to a literature review, these outcomes were comparable to those of simultaneous open hepatic resection and splenectomy.

**Conclusions:**

Laparoscopic-guided radio-frequency ablation with laparoscopic splenectomy and endoscopic variceal ligation could be an available technique for patients with HCC <3 cm, hypersplenism, and esophagogastric varices. This approach may help to minimize the surgical risks and results in a fast increase in platelet counts with an acceptable rate of complications.

## Background

Hepatocellular carcinoma (HCC) is one of most common cancers in China [1]. The treatment of HCC is complicated and challenging due to the frequent presence of hepatic cirrhosis and portal hypertension, which may result in coagulation dysfunction, esophagogastric varices, anemia, abdominal collaterals, and peripheral cytopenia, particularly thrombocytopenia [2–5].

Therefore, treatment for HCC should remove the tumor with minimal invasiveness. Cirrhotic hypersplenism can result in peripheral cytopenia. Because cytopenia is reversible after splenectomy, splenic embolism or resection seems to be an appropriate choice. Simultaneous hepatectomy and splenectomy are associated with improved 5-year tumor-free survival in patients with HCC and hypersplenism [6], and laparoscopic splenectomy (Lap-sp) has recently become a potential choice [7]. Moreover, radio-frequency ablation (RFA) has been reported to have the same outcomes as liver resection for patients with tumors <3 cm [8–10].

In an animal model, splenectomy improved the status of hepatic cirrhosis [11] and helped hepatic recovery, promoting liver regeneration after massive liver resection [12]. Recently, some studies reported that liver function was improved after splenectomy in patients with portal hypertension and hypersplenism.

Therefore, the objective of this study was to investigate the effects of laparoscopic-guided RFA with Lap-sp and endoscopic variceal ligation in patients with HCC <3 cm, cirrhosis, and esophagogastric varices.

## Methods

### Patients

This was a retrospective study carried out in 25 patients with HCC, hypersplenism, and esophagogastric varices and who underwent simultaneous laparoscopic-guided ablation and Lap-sp with endoscopic variceal ligation between January 2012 and October 2014 at the Department of General Surgery at the Third Affiliated Hospital of Sun Yat-sen University (Guangzhou, China). Informed contents were accepted and signed off by all patients and their family members before surgery. The study was approved by the Committee of Ethics of the Third Affiliated Hospital of Sun Yat-sen University. Written informed consent was obtained from the patient for the publication of this report and any accompanying images.

At our center, selection criteria for this surgical approach were (1) 18–70 years old; (2) diagnosis of HBV- or HCV-related liver cirrhosis with portal hypertension; (3) spleen thickness >4.1 cm; (4) HCC ≤3 cm; (5) first surgical attempt; (6) severe esophagogastric varicosity confirmed by gastroscopy; (7) Child-Pugh class A or B, score ≤9; and (8) platelets ≤50 × 10^9^/L and leukocytes ≤3.5 × 10^9^/L. This approach was not proposed to patients in case of (1) other tumors; (2) HIV-positive test; (3) any immunodeficiency or autoimmune disease (e.g., rheumatic arthritis, Buerger’s disease, multiple sclerosis, type 1 diabetes); (4) any organ failure; or (5) mental illness.

### Surgical procedure

Surgery was performed under general anesthesia with the patient in the right semi-decubitus position. Five trocars with a diameter of 5 mm/12 mm were introduced into the abdominal cavity through the left upper quadrant of the abdomen. The abdominal cavity was insufflated with 13–15 mmHg of CO_2_, and a 30° laparoscope was inserted. Mobilization of the spleen was performed using the Ligasure vessel sealing system and an ultrasonic scalpel. The tissues around the splenic hilum including the splenic arteries and veins were cut using an autosuture device, and the spleen was freed. The spleen was packed in a plastic sac and cut into pieces using scissors through one of the trocar ports. The fragmented spleen was then removed with the sac from the abdomen without extending the wound. Next, the RFA needle was inserted into the center of the target HCC nodule by the radiologist. RFA was applied continuously for 8–12 min. The endpoint of RFA was determined mainly according to ultrasonography of an index tumor that was fully covered by the hyperechoic ablated zone. Finally, endoscopic ligation was performed as classically described [13]. All patients were treated by the same surgical team. An empirical course of antibiotics was started 30 min before surgery and was later adjusted based on the results of bacterial cultures. The duration was 48 h on cases of negative results.

### Follow-up

Blood routine examination, liver function, and color Doppler ultrasound were performed on days 3, 7, 14, and 30, months 2 and 3, and then every 3 months. Virology, CT, and gastroscopy were performed every 3 months after surgery. Follow-up was censored in October 2014 or at the time of death.

### Statistical analysis

Statistical analysis was performed with SPSS 20.0 (IBM, Armonk, NY, USA). Continuous variables are presented as mean ± standard deviation. Categorical variables are presented as frequencies. The Kaplan-Meier method was used to present overall survival (OS) and disease-free survival (DFS). DFS was defined as the time from initial treatment to the first evidence of recurrence. Survival was censored at the last follow-up. Two-tailed *P* values <0.05 were considered significant.

## Results

### Characteristics of the patients

This study included 24 men and 1 woman (mean age 51.5 ± 15.0 years). All patients had symptoms of hepatic cirrhosis and portal hypertension, and HBV infection (21/25) was the main cause, followed by HCV (3/25) and co-infection (1/25). Alcohol abuse history was present in ten patients. Mean tumor size was 20.0 ± 4.6 mm (range 13–28 mm) and spleen thickness was >4.1 cm and length was 18.4 ± 2.5 cm (range 14–22 cm (Table 1).

**Table 1:** Baseline characteristics of the patients

Characteristics	Values (*n* = 25)
Age (mean ± SD)	51.52 ± 15.03
Gender (male/female)	24/1
Portal hypertension	25
Etiology of cirrhosis	
HBV	21
HCV	3
HBV + HCV	1
Alcoholic	10
Child-Pugh classification	A 20 (80 %)
	B 5 (20 %)
Serum AFP (μg/L)	
≤20	12 (48 %)
20–400	7 (28 %)
>400	6 (24 %)
Tumor number	1 (*n* = 25)
Tumor size (mm)	20 ± 4.56
≤20	17 (68 %)
20–30	8 (32 %)
Mean HBV DNA load (log10 IU/mL)	3.10 ± 1.59
Platelet count (×10^9^/L) (mean ± SD)	31.16 ± 6.92
White blood cell count (×10^9^/L) (mean ± SD)	3.81 ± 0.62
ALT (IU/L) (mean ± SD)	41.36 ± 33.76
AST (IU/L) (mean ± SD)	39.80 ± 24.91
Albumin (g/L) (mean ± SD)	41.17 ± 3.68
Bilirubin (μmol/L) (mean ± SD)	15.36 ± 4.99
Length of spleen (cm)	18.36 ± 2.46

*AFP* α-fetoprotein, *HBV* hepatitis B virus, *HCV* hepatitis C virus, *ALT* alanineaminotransferase, *AST* aspartate aminotransferase, *SD* standard deviation

### Perioperative characteristics

No cases had to be converted to an open procedure. All patients tolerated the operations well without major intraoperative complications (Table 2).

**Table 2:** Intraoperative and postoperative complications

Complications	Number	Percent
Converted to open surgery	0	0
Hemorrhage	0	0
Bile leakage	0	0
Infection of port(s)	0	0
Hepatic encephalopathy	0	0
Hepatorenal syndrome	0	0
Postoperative deterioration in liver function tests	2	8
Postoperative worsening of ascites	3	12
Portal vein thrombosis	5	20

### Postoperative outcomes

No patient had a positive bacterial culture test or became febrile. Portal vein thrombosis occurred in five patients. Three patients had worsening ascites after surgery, two of whom showed increases in bilirubin levels, manifesting the deterioration in liver function. The mean length of stay was 7.0 ± 1.5 days (range 5–15 days). The mean costs of hospitalization were 8064 USD.

### Changes in blood cell counts

**Fig. 1:**
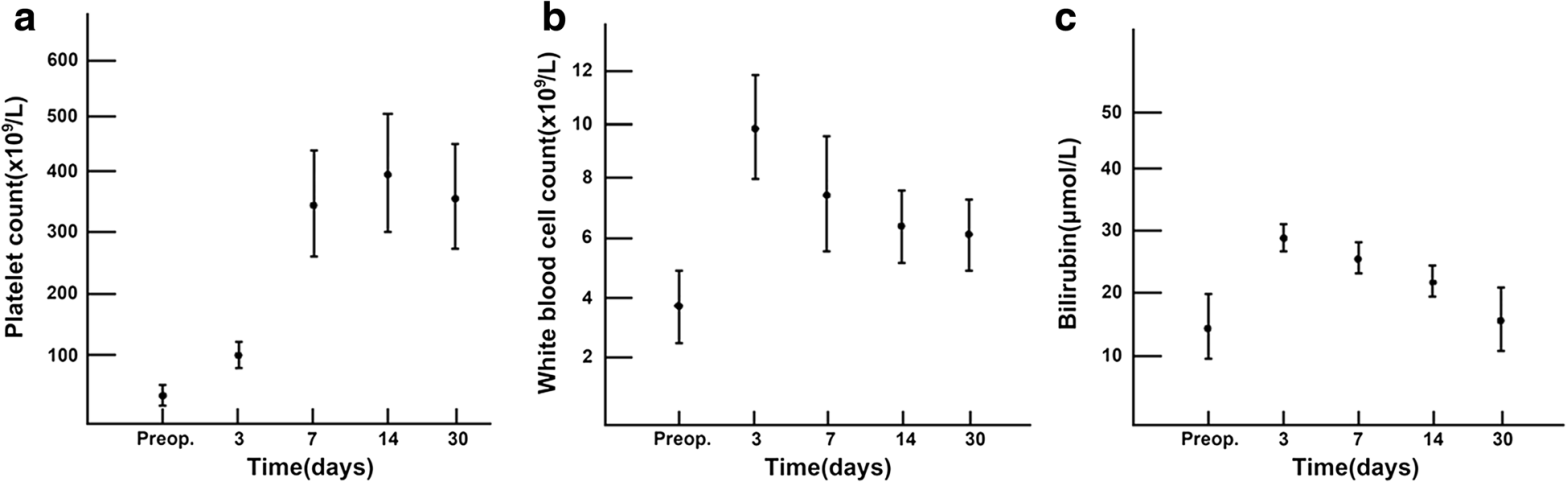
Preoperative and postoperative platelet counts (**a**), white blood cell counts (**b**), and total bilirubin levels (**c**)

The average platelet counts were 31.16, 101.02, 334.52, 378.34, and 349.78 × 10^9^/L before surgery and on days 3, 7, 14, and 30, respectively (*P* < 0.05). White blood cell counts also showed the same trend (3.81, 9.56, 7.23, 6.68, and 6.15 × 10^9^/L, respectively). In addition, the mean total bilirubin levels were 15.36, 28.92, 25.42, 21.03, and 16.44 μmol/L, respectively. These parameters changed during the first month and then remained stable (Fig. 1).

### Follow-up

**Fig. 2:**
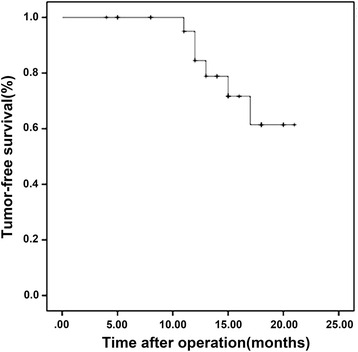
Kaplan-Meier curve of disease-free survival

All patients were followed up (1–21 months). Six patients had HCC recurrence (11–17 months), and one patient died of HCC 14 months after surgery. DFS at 21 months was 61.4 % in all patients (Table 3 and Fig. 2).

**Table 3:** Recurrence, overall survival, and disease-free survival

Patients (*n* = 25)	Percent
Recurrence	24
Overall survival	
1 year	100
21 months	96
Disease-free survival	
1 year	78.8
21 months	61.4

## Discussion

Recently, some studies reported that liver function was improved after splenectomy in patients with portal hypertension and hypersplenism. A study by Shimada et al. [14] suggested improvements in Child-Pugh scores after splenectomy, while Imura et al. [15] demonstrated an additional improvement in nutritional metabolism after splenectomy.

Because the residual splenic tissue remaining after partial embolism may regenerate [16], spleen embolism has not been demonstrated to decrease the total bilirubin levels and may not relieve hypersplenism sufficiently to improve liver function [17, 18]. In addition, the timing of splenectomy is controversial. Hanazaki et al. [19] and Shimada et al. [14] suggested that splenectomy should be performed first, followed by hepatectomy, because portal vein thrombosis occurs frequently after synchronous splenectomy and increases the morbidity of the procedure. Meanwhile, Sugawara et al. [17] suggested that when HCC is located at the left lobe and superficial region of the liver, synchronous splenectomy and liver resection are beneficial because the left lobe is located near the spleen, making the hepatectomy procedure much easier. For tumors located at the right lobe of the liver, they suggested staged splenectomy first and hepatectomy 30 to 58 days later [17].

Previous studies showed the feasibility of synchronous splenectomy and liver resection without compromising perioperative safety [17, 18, 20–22]. Chen et al. [6] reported that synchronous open hepatectomy and splenectomy were associated with an improved 5-year DFS.

Synchronous laparoscopic-guided RFA with Lap-sp and endoscopic variceal ligation is safe and feasible, not only minimizing invasiveness but also avoiding spleen regeneration. In this series, no hemorrhage or conversion occurred during the perioperative period. In the previous studies, the mean operative time and blood loss were 103–305 min and 380–1300 mL, respectively [6, 17, 18, 20–22]. In the current study, they were 128 ± 18 min and 206 ± 57 mL, respectively, suggesting that this approach could significantly reduce both parameters. Patients also had a shorter length of stay without compromising safety (7 vs. 13.2 days) [18].

The frequency of portal vein thrombosis after splenectomy has been reported to range from 2 to 48 % in patients with cirrhosis or portal hypertension [23], and 50 % in non-cirrhotic patients [24]. The prevalence of operative mortality from portal vein thrombosis after splenectomy ranged from 0 to 18 % [17, 19], compared to 20 % in this present study. The diameter of the splenic vein, low white blood cell counts, and spleen weight were reported as being independent risk factors for portal vein thrombosis [25]. Regarding portal vein thrombosis, the risk factors should be more strictly evaluated, managed, and treated in a future study.

High bilirubin levels secondary to hypersplenism is caused by an increase in bilirubin production, which overloads the capacity of the liver to metabolize bilirubin [26]. Splenectomy might contribute to the decrease of total bilirubin levels, but it is difficult to demonstrate any direct contribution, and postoperative care to protect liver function could also have some influence. Splenectomy has been suggested for the treatment of secondary hypersplenism and thrombocytopenia [27]. Some investigators have carried out splenectomy for patients who had undergone liver transplantation complicated by persistent thrombocytopenia [28]. Takayama et al. [26] and Sugawara et al. [17] used this procedure to extend the patient selection criteria for HCC resection in cirrhotic patients.

Previous studies showed that the 1-year survival of patients who underwent simultaneous liver resection and splenectomy is 82–90 % [6, 17, 18, 20–22] and that the 1-year DFS is 80 % [6, 22], which are similar to this present series. However, in the study by Chen et al. [6], the 5-year DFS was significantly higher. Future studies may help to clarify these issues.

This study was a retrospective and uncontrolled pilot study comparing using previously reported results as comparator. Prospective studies of Lap-sp and splenectomy for HCC patients are necessary.

## Conclusions

In conclusion, laparoscopic-guided ablation with Lap-sp and endoscopic variceal ligation could be an elective technique for patients with HCC <3 cm, hypersplenism, and esophagogastric varices. The approach seems to minimize the risks and to result in a fast recovery of the platelet count with an acceptable rate of complications. Although the long-term outcomes after this procedure remain to be determined, future randomized controlled prospective studies are needed to confirm these findings.

## Abbreviations

DFS, disease-free survival; HCC, hepatocellular carcinoma; Lap-sp, laparoscopic splenectomy; OS, overall survival; RFA, radio-frequency ablation
